# Causal effect of gut microbiota of *Defluviitaleaceae* on the clinical pathway of “Influenza–Subacute Thyroiditis–Hypothyroidism”

**DOI:** 10.3389/fmicb.2024.1354989

**Published:** 2024-02-26

**Authors:** Xin Zhang, Pei-Heng Li, Dongyue Wang, Hancong Li, Xiangyu Kong, Gongshuang Zhang, Yue Zhao, Jiaye Liu, Wenshuang Wu, Yuwei Zhang, Zhi-Hui Li, Han Luo

**Affiliations:** ^1^Department of Radiation Oncology, Cancer Center, West China Hospital, Sichuan University, Chengdu, China; ^2^Department of Biotherapy, Cancer Center, West China Hospital, Sichuan University, Chengdu, China; ^3^Division of Thyroid Surgery, Department of General Surgery, West China Hospital, Sichuan University, Chengdu, Sichuan, China; ^4^Department of Ophthalmology, West China Hospital, Sichuan University, Chengdu, China; ^5^Department of Endocrinology and Metabolism, West China Hospital of Sichuan University, Chengdu, China; ^6^Center for Diabetes and Metabolism Research, West China Hospital of Sichuan University, Chengdu, China; ^7^Department of Laboratory Medicine/Research Centre of Clinical Laboratory Medicine, West China Hospital, Sichuan University, Chengdu, Sichuan, China

**Keywords:** gut microbiota, hypothyroidism, influenza, Mendelian randomization, subacute thyroiditis

## Abstract

**Introduction:**

Hypothyroidism has been found to be influenced by gut microbiota. However, it remains unclear which a taxon of gut microbiota plays a key role in this function. Identifying the key bacteria affects hypothyroidism and through what mechanism will be helpful for the prevention of hypothyroidism through specific clinical pathways.

**Materials and methods:**

In Study A, 35 families and 130 genera of gut microbiota are used as exposures, with hypothyroidism as the outcome. The causal effect of the gut microbiota on hypothyroidism is estimated through two-sample Mendelian randomization. Combining the results of the two taxonomical levels, key taxa are selected, which in Study B are investigated for their causal association with multiple generally admitted causes of hypothyroidism and their more upstream factors. For validating and revealing the potential mechanism, enrichment analyses of the related genes and interacting transcription factors were performed.

**Results:**

In Study A, *Defluviitaleaceae* (OR: 0.043, 95% CI: 0.005–0.363, *P* = 0.018)/*Defluviitaleaceae_UCG_011* (OR: 0.385, 95% CI: 0.172–0.865, *P* = 0.021) are significantly causally associated with hypothyroidism at both taxonomical levels. In Study B, *Defluviitaleaceae* family and *Defluviitaleaceae_UCG_011* genus show the causal association with decreased thyroiditis (Family: OR: 0.174, 95% CI: 0.046–0.653, *P* = 0.029; Genus: OR: 0.139, 95% CI: 0.029–0.664, *P* = 0.043), decreased subacute thyroiditis (Family: OR: 0.028, 95% CI: 0.004–0.213, *P* = 0.007; Genus: OR: 0.018, 95% CI: 0.002–0.194, *P* = 0.013), decreased influenza (Family: OR: 0.818, 95% CI: 0.676–0.989, *P* = 0.038; Genus: OR: 0.792, 95% CI: 0.644–0.974, *P* = 0.027), and increased anti-influenza H3N2 IgG levels (Family: OR: 1.934, 95% CI: 1.123–3.332, *P* = 0.017; Genus: OR: 1.675, 95% CI: 0.953–2.943, *P* = 0.073). The results of the enrichment analysis are consistent with the findings and the suggested possible mechanisms.

**Conclusion:**

*Defluviitaleaceae* of the gut microbiota displays the probability of causally inhibiting the clinical pathway of “Influenza–Subacute Thyroiditis–Hypothyroidism” and acts as the potential probiotics to prevent influenza, subacute thyroiditis, and hypothyroidism.

## Graphical Abstract

This study indicates that *Defluviitaleaceae* in the gut microbiota displays the probability of causally inhibiting the clinical pathway of “Influenza–Subacute Thyroiditis–Hypothyroidism.” 
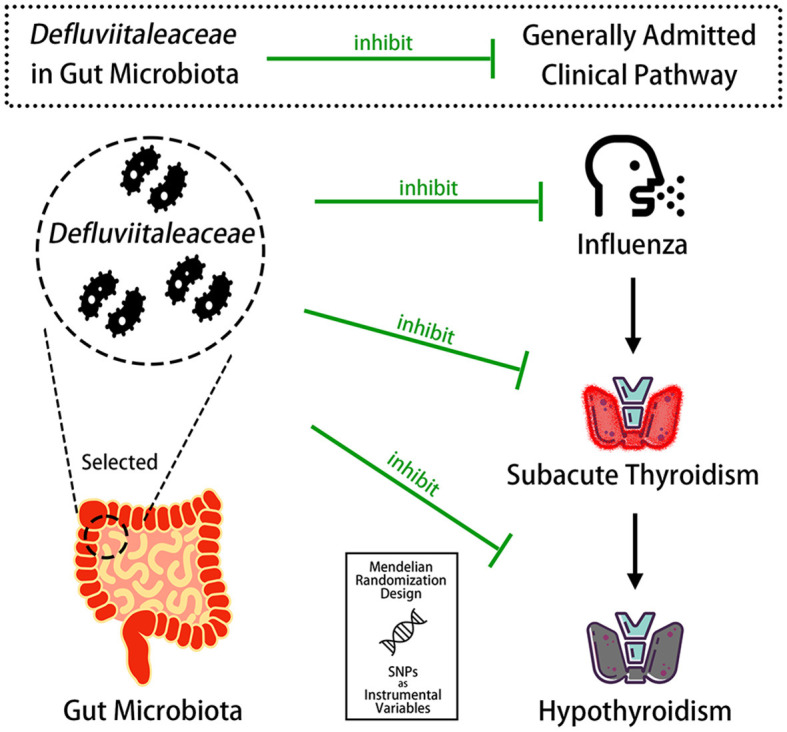

## Introduction

Hypothyroidism is defined as the condition of thyroid hormone deficiency. The prevalence of hypothyroidism is approximately 3%−7% (Hollowell et al., 2002; Garmendia Madariaga et al., 2014). Patients can suffer from fatigue, lethargy, cold intolerance, weight gain, constipation, change in voice, and dry skin. The clinical manifestations range from life-threatening conditions to no symptoms at all (Chaker et al., 2017). Hypothyroidism has also been found to be associated with heart diseases, such as the coronary artery disease and heart failure, and cause higher mortality (Vanhaelst et al., 1967; Rodondi et al., 2010; Gencer et al., 2012). Hypothyroidism can be classified into primary hypothyroidism, central hypothyroidism, and peripheral hypothyroidism. Among them, thyroiditis, such as chronic autoimmune thyroiditis and subacute thyroiditis, are the common causes (Chaker et al., 2017). The standard treatment of hypothyroidism is through thyroid hormone replacement therapy with levothyroxine. However, some patients fail to improve their quality of life after the treatment (Hegedüs et al., 2022). Thus, it is important to understand the cause of hypothyroidism and identify the potential therapies to prevent and treat hypothyroidism.

Human gut microbiota is composed of trillions of microorganisms, including bacteria, fungi, viruses, and archaea (Eckburg et al., 2005). As an essential component in human body, gut microbiota takes part in the digestion of nutrients, the metabolism of several drugs, and host immune system development, which are pivotal for human body homeostasis (Round and Mazmanian, 2009; Dodd et al., 2017). The development of *16S rRNA* gene and metagenome sequencing have led to studies on the interactions between microbiota and humans into a new era (Gill et al., 2006; Qin et al., 2010). Numerous studies have been performed to explore the relationship between intestinal microbiota and diseases, such as gastrointestinal diseases, psychiatric diseases, metabolic diseases, and autoimmune diseases (Tlaskalová-Hogenová et al., 2011; Chen et al., 2016; Vitale et al., 2021). Some studies have indicated the association between gut microbiota and thyroid diseases, along with the concept of “thyroid–gut–axis” (Lerner et al., 2017; Fröhlich and Wahl, 2019; Jiang et al., 2022; Virili et al., 2023). However, whether the alteration of microbiota is the cause of thyroid diseases cannot be determined. Taking advantage of Mendelian randomization (MR), the causal relationship between exposure and outcome can be ascertained. Compared with traditional observational studies, MR utilizes genetic variations to infer the causal connection, which is more reliable (Yao et al., 2022).

This study is designed to determine the causal effect of gut microbiota on hypothyroidism and further explore how the screened taxon of microbiota causes hypothyroidism.

## Materials and methods

### Study design

This study is designed to determine the causal effect of gut microbiota on hypothyroidism and further explore through which pathway the screened taxon of microbiota causes hypothyroidism. MR analyses (Burgess and Thompson, 2021) are used to provide evidence for the causal association between microbiota and steps in the clinical pathway to hypothyroidism.

**Figure 1:**
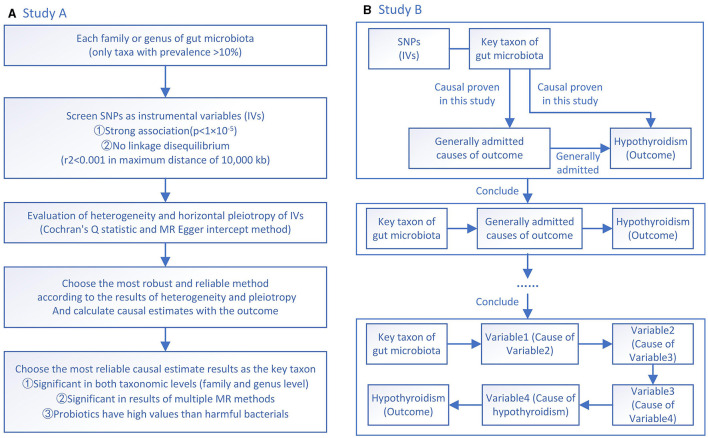
**(A)** A flowchart of Study A investigating the causal effects between gut microbiota and hypothyroidism; **(B)** A flowchart of Study B investigating how *Defluviitaleaceae* family and *DefluviitaleaceaeDefluviitaleaceae_UCG_011* genus affect factors toward hypothyroidism.

Study A is designed to screen the potential taxa of the gut microbiota that are causally related to hypothyroidism (Figure 1A). In Study A, two families and genera of gut microbiota that can causally affect the occurrence of hypothyroidism are investigated. Since the rigorous correction for multiple comparisons might neglect potential taxa that are causally associated with hypothyroidism, multiple testing is not performed in Study A. Screening is done according to three criteria. The first criterion is significant at both family and genus levels, which can increase the robustness and reliability of the results of the selected microbiota. Although it might wrongly remove some potentially valuable microbiota, it can better make sequent results correct. The second criterion is better to be validated by multiple MR methods. Each MR method has its own strengths. With significant results of more MR methods, the sequent analyses become more reliable. The third criterion is, if there are probiotics and harmful microbiota screened at the same time, the probiotics will be chosen as the final candidate target. Because the supplement of a bacteria is easier and more economical than targeting a bacterium, the findings will have higher clinical values. The screened key taxon of the gut microbiota is then used in Study B to determine more specific mechanisms.

In Study B, the screened “key taxon” is used to investigate whether it has a causal effect on known causative factors of hypothyroidism with MR analyses (Figure 1B). Then, the former known causative factors are further investigated to identify a causally affected clinical pathway. By confirming the linear causal effect of “key taxon” at each step of the known clinical pathway, the application potential of the identified taxon of gut microbiota can be proven.

### Data source

All data used in this study are summary-level data of genome-wide association studies (GWAS). The source of all data is summarized in Supplementary Table S1.

The data of gut microbiota are available from the MiBioGen database (https://mibiogen.gcc.rug.nl/), which was provided by Kurilshikov et al. (2021). These comprehensive GWAS data of gut microbiota were collected from a pool of 18,340 individuals from 24 cohorts. The data of 165 taxa (including 35 families and 130 genera) are finally used in the present study. The details about the measurement methods can be checked in previously published studies by Kurilshikov et al. (2021). Supplementary Table S2 lists all the taxa of gut microbiota used in this study (in the column “exposure”).

Fecal samples were collected from 18,340 people, the majority of whom are Europeans with a median age of approximately 55 years. After fecal sample collection from each cohort, DNA purification kits were used to extract the DNA. The MiSeq technology was used for DNA sequencing in most cohorts, while the HiSeq2500 technology was used for one cohort. Due to differences in the *16S rRNA* gene domains among the cohorts, the author directly mapped the reads to the SILVA ribosomal RNA database for taxonomic classification. A cutoff posterior probability of 0.8 was applied at each taxonomic level to assign reads to their respective taxa. This posterior cutoff probability was assessed independently for each taxonomic level. Only taxa present in over 10% of the samples within each cohort were left. For the comprehensive mapping of multi-cohort Quantitative Trait Loci, the study-wide criteria were an effective sample size of a minimum of 3,000 samples and presence in at least three cohorts. The single nucleotide polymorphism (SNP) panels of cohorts were mainly provided by Illumina and Affymetrix. After quality control, SNPs strongly associated with one of the different taxa (*P* < 0.0001), as well as their corresponding positions, alleles, regression results, and sample size, all of which were recorded and uploaded to the MiBioGen database used in this study.

The outcome variables are all collected from the IEU OpenGWAS project (https://gwas.mrcieu.ac.uk/), which was built by the University of Bristol, and contains summary-level GWAS data from multiple databases. For example, the outcome variable of Study A, hypothyroidism, is derived from UK Biobank but downloaded from IEU OpenGWAS project.

In the investigation of factors contributing to hypothyroidism, the majority of data are derived from FinnGen database, which is built by the University of Helsinki, since it divided hypothyroidism into different subtypes, such as autoimmune hypothyroidism and hypothyroidism due to medicaments and other exogenous factors. However, in the exploration of infectious variables related to subacute thyroiditis, there are no data about coxsackievirus and adenovirus in the FinnGen database. Instead, the summary-level GWAS data of coxsackievirus and adenovirus receptor provided by Sun et al. (2018) are used. Details can be checked in each source database (Supplementary Table S1).

### Selection of instrument variables

The instrument variable (IV) selection is done according to the standardized processes of MR analyses using *P-*value threshold and linkage disequilibrium (LD) clumping. To obtain the adequate number of genetic variations as IVs and retain IVs strongly associated with each taxon of gut microbiota, the significant threshold is finally set at 1 × 10^−5^, which is also a common threshold in many high-quality MR studies of gut microbiota (Li et al., 2022; Song et al., 2023). Then, SNPs with LD are also removed using a threshold of r^2^ < 0.001 and a maximum distance of 10,000 kb. Finally, 2,105 independent SNPs associated with 165 bacterial traits are identified as IVs.

### Mendelian randomization analysis methods

This study adhered to the three core assumptions of MR, as outlined by Bowden and Vansteelandt (2011): (1) the IVs are strongly associated with the taxa of gut microbiota, (2) IVs are independent of confounders affecting outcome variables, and (3) the IVs affect the outcome variable solely through the exposure and not via alternative pathways. The first assumption is met in the selection of SNPs, while the last two are assessed by pleiotropy tests.

In this study, five MR analytical methods are used, namely, inverse variance-weighted (IVW), MR Egger, weighted median, weighted mode, and simple median. IVW is the method with the greatest statistical power but biased if there is horizontal pleiotropy. On the contrary, the MR Egger method is robust to horizontal pleiotropy. The weighted median method is more robust to outliers than IVW and useful when significant result is found in the heterogeneity test. The weighted mode and simple median methods are supplementary to the weighted median method. Therefore, the primary methods of MR analyses are IVW when no horizontal pleiotropy or heterogeneity is found, MR Egger when there is horizontal pleiotropy, and weighted median when there is heterogeneity but no horizontal pleiotropy (Burgess and Thompson, 2021). The Supplementary material contains more detailed introduction of these analytical methods of MR and the way and rationale for the method selection.

MR analysis relies on Mendel's laws of inheritance and instrumental variable estimation methods, which are designed to calculate causal effects in the presence of unobserved confounding (Sanderson et al., 2022). To address potential confounders, heterogeneity and pleiotropy have to be estimated and used for the selection of the robust MR methods. Heterogeneity refers to differences and outliers in SNP-specific causal estimates. Cochran's *Q* statistic is calculated to evaluate heterogeneity in this study, which is a weighted sum of the squared distances of the SNP-specific causal estimates from the overall IVW estimate. The term pleiotropy is sometimes used interchangeably with “horizontal pleiotropy,” while “mediation” is sometimes called “vertical pleiotropy.” Horizontal pleiotropy indicates that IVs can affect outcomes through covariate rather than only through the exposure, while vertical pleiotropy indicates exposure affect outcome via the covariate. Horizontal pleiotropy is detected by the MR Egger method, while horizontal pleiotropy can also be detected robustly.

In addition, sensitivity analyses with the leave-one-out method are performed to assess the reliability, stability, and robustness of the causal estimates, specifically investigating whether the results were disproportionately influenced by any individual SNP. The calculation of all analyses was done with the “TwoSampleMR” package (version 0.5.6) on R (version 4.2.2). The results of MR analyses are presented with odds ratio (OR) and confidence intervals (CIs). With a significance level below *P* of < 0.05, it can be concluded that there might be a causal relationship.

### Exploration of related mechanisms with enrichment analysis

Since the MR analysis of clinical outcomes does not reveal the molecular mechanisms, we attempted to carry out enrichment analyses of the key taxon IVs, although these results might be only suggestive rather than confirmed.

First, the single nucleotide polymorphism database (dbSNP) provided by the National Institutes of Health of the United State was utilized to determine the related genes of IVs (https://www.ncbi.nlm.nih.gov/snp/). Then, the tool known as the database for annotation, visualization and integrated discovery (DAVID) provided by the National Institutes of Health of the United States was used to perform gene-enrichment analysis with annotation categories of diseases (from Genetic Association Disease Database) and gene ontology (Huang and Sherman, 2009; Sherman et al., 2022) (https://david.ncifcrf.gov/). The default threshold used is *P* < 0.1 and count >2.

We attempted to use the enrichment analysis of the Kyoto Encyclopedia of Genes and Genomes (KEGG) pathways, but it produced no significant results. As an alternative, the transcription factor genes that interact with the products of IV-related genes were first investigated to indirectly explore the possible related molecular pathways. The interacting transcription factors were also investigated using the DAVID tool. Then, the enrichment analysis of KEGG pathways was performed again with the identified transcription factors to ascertain the possible molecular pathways involved (It is worth noting that these indirect results are suggestive and have much weaker power).

## Results

### Study A: effect of gut microbiota on hypothyroidism

A total of 2,151 SNPs were selected as IVs for 35 families and 130 genera of gut microbiota, which is listed in Supplementary Table S2. Five MR methods mentioned before were performed for each taxon, and all results are displayed in Supplementary Table S3, while the results of tests of heterogeneity and horizontal pleiotropy are listed in Supplementary Table S4. The directions of each pair of taxon and outcome were further confirmed with the Steiger test to avoid reverse causation (Supplementary Table S6).

**Figure 2:**
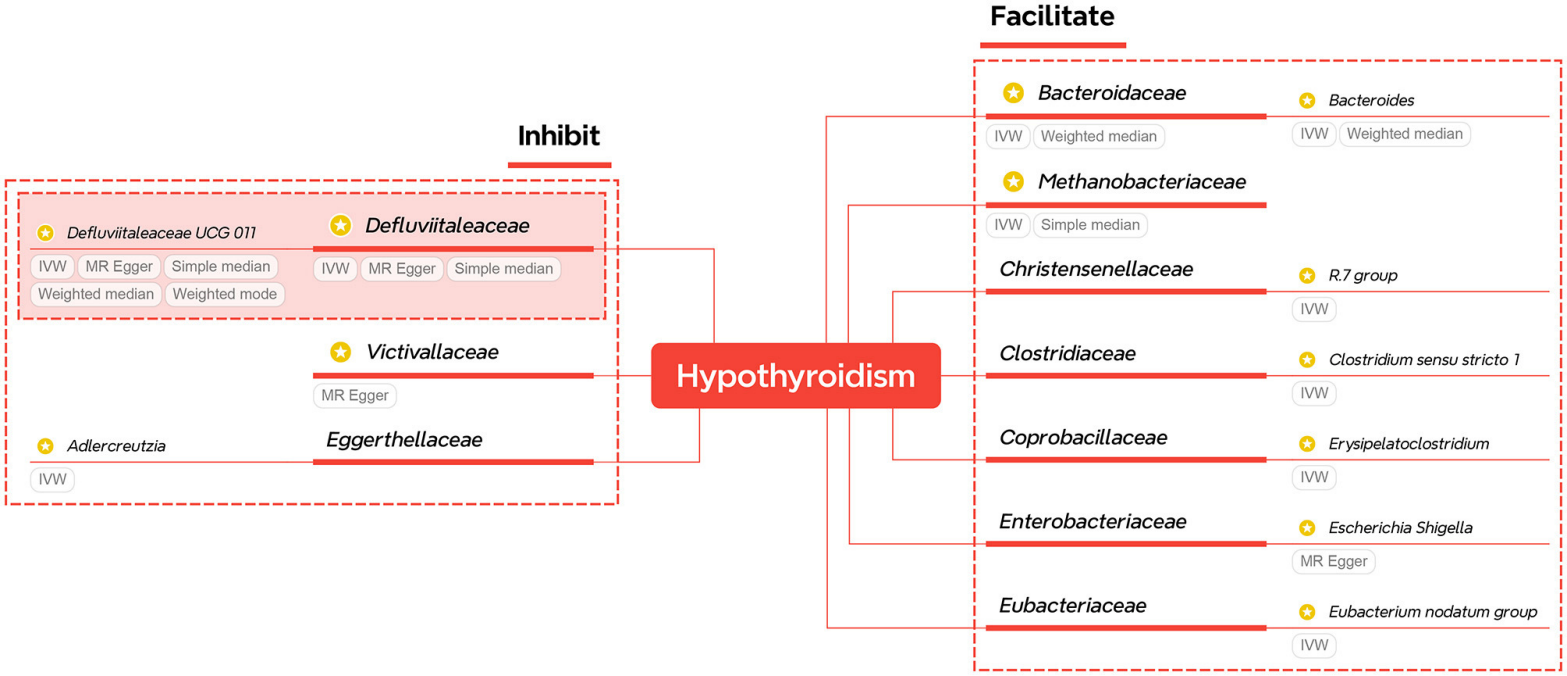
A summary of significant findings of Study A. The taxa with yellow-star marks have statistical significance of primary methods.

For easy check, the significant results of primary methods are summarized and displayed in Table 1, while the corresponding results of heterogeneity and horizontal pleiotropy are listed in Supplementary Table S5. The relationships of significant families and genera are shown in Figure 2. The figure shows that two families and two genera can causally inhibit hypothyroidism, while two families and six genera can facilitate hypothyroidism. Only *Defluviitaleaceae* (OR 0.043, 95%CI 0.005–0.363, *P* = 0.018)/*Defluviitaleaceae_UCG_011* (OR 0.385, 95%CI 0.172–0.865, *P* = 0.021) and *Bacteroidaceae* (OR 3.363, 95%CI 1.312–8.617, *P* = 0.012)/*Bacteroides* (OR 3.363, 95%CI 1.312–8.617, *P* = 0.012) are significant at both the taxonomical levels.

**Table 1:** The significant causal estimates of gut microbiota with hypothyroidism in MR analyses.

**Primary method**	**N_SNP**	**Beta**	**SE**	***P-*value**	**OR**	**Lower limit**	**Higher limit**
Inverse variance weighted	9	1.213	0.480	0.012	3.363	1.312	8.617
MR Egger	11	−3.155	1.093	0.018	0.043	0.005	0.363
Inverse variance weighted	10	0.477	0.230	0.038	1.611	1.026	2.529
MR Egger	12	−2.112	0.903	0.042	0.121	0.021	0.711
Inverse variance weighted	11	0.395	0.180	0.028	1.484	1.043	2.112
Inverse variance weighted	8	−0.755	0.321	0.019	0.470	0.251	0.882
Inverse variance weighted	9	1.213	0.480	0.012	3.363	1.312	8.617
Inverse variance weighted	10	0.815	0.413	0.048	2.260	1.007	5.074
Inverse variance weighted	7	0.672	0.302	0.026	1.959	1.085	3.537
Inverse variance weighted	9	−0.954	0.413	0.021	0.385	0.172	0.865
Inverse variance weighted	15	0.624	0.250	0.013	1.866	1.143	3.046
MR egger	10	2.986	1.018	0.019	19.816	2.693	145.812

Although the primary method results of both pairs are significant, more methods of *Defluviitaleaceae* also support significance. Meanwhile, a supplementary of probiotic bacteria is more economical and has higher application value than targeting harmful bacteria. Therefore, the *Defluviitaleaceae* family and *Defluviitaleaceae_UCG_011* genus are selected as key taxa of gut microbiota for further investigation in Study B.

### Study B: effect of *Defluviitaleaceae* on causes toward hypothyroidism

In this part, SNPs used as IVs is listed in Supplementary Table S7. All of the MR results in this part are summarized in Supplementary Table S8, while results of tests of heterogeneity and horizontal pleiotropy are listed in Supplementary Table S9. The directions of causation between *Defluviitaleaceae* and outcomes are checked with Steiger test, and there is no reverse causation (Supplementary Table S11). The MR results of outcomes, which contain significant results are listed in Table 2 for easy check, and corresponding results of heterogeneity and pleiotropy are in Supplementary Table S10.

**Table 2:** The significant causal estimates of *Defluviitaleaceae* family and *DefluviitaleaceaeDefluviitaleaceae_UCG_011* genus with hypothyroidism, thyroiditis, sub thyroiditis, influenza, and anti-influenza H3N2 IgG level in MR analyses.

**Outcome**	**Exposure**	**Primary method**	**N_SNP**	**Beta**	**SE**	***P* value**	**OR**	**Lower Limit**	**Higher Limit**
Hypothyroidism	genus.DefluviitaleaceaeUCG011.id.11287	Inverse variance weighted	9	−0.954	0.413	0.021	0.385	0.172	0.865
Hypothyroidism	family.Defluviitaleaceae.id.1924	MR Egger	11	−3.155	1.093	0.018	0.043	0.005	0.363
Thyroiditis	genus.DefluviitaleaceaeUCG011.id.11287	MR Egger	9	−1.973	0.798	0.043	0.139	0.029	0.664
Thyroiditis	family.Defluviitaleaceae.id.1924	MR Egger	11	−1.749	0.675	0.029	0.174	0.046	0.653
Subacute thyroiditis	genus.DefluviitaleaceaeUCG011.id.11287	MR Egger	9	−4.008	1.208	0.013	0.018	0.002	0.194
Subacute thyroiditis	family.Defluviitaleaceae.id.1924	MR Egger	11	−3.587	1.040	0.007	0.028	0.004	0.213
All influenza	genus.DefluviitaleaceaeUCG011.id.11287	Inverse variance weighted	9	−0.233	0.105	0.027	0.792	0.644	0.974
All influenza	family.Defluviitaleaceae.id.1924	Inverse variance weighted	11	−0.201	0.097	0.038	0.818	0.676	0.989
Anti-Influenza virus subtype H3N2 IgG levels	genus.DefluviitaleaceaeUCG011.id.11287	Inverse variance weighted	8	0.516	0.288	0.073	1.675	0.953	2.943
Anti-Influenza virus subtype H3N2 IgG levels	family.Defluviitaleaceae.id.1924	Inverse variance weighted	9	0.660	0.277	0.017	1.934	1.123	3.332

**Figure 3:**
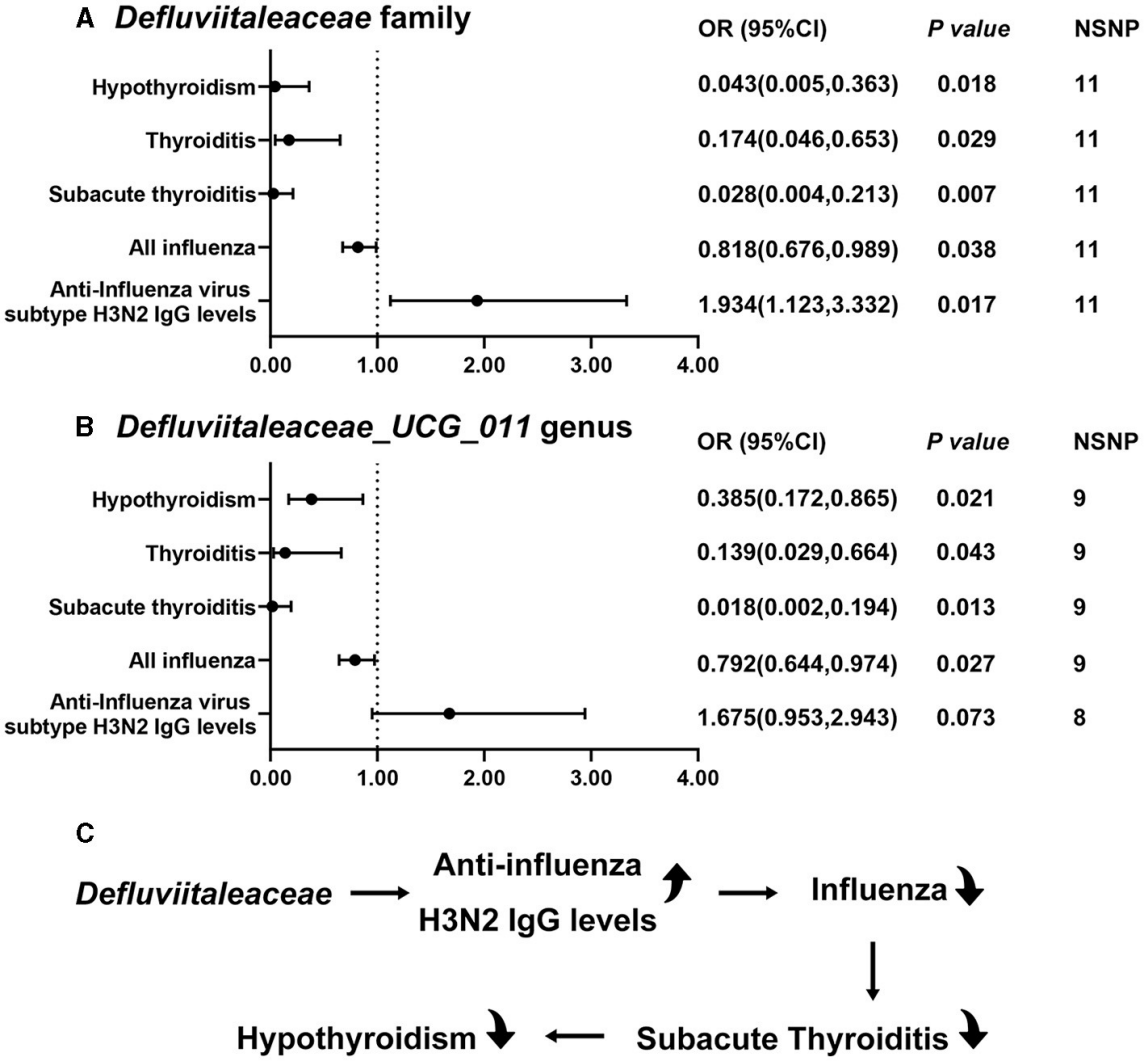
Significant MR results of *Defluviitaleaceae* family and *DefluviitaleaceaeDefluviitaleaceae_UCG_011* genus. **(A)** The results of *Defluviitaleaceae* family; **(B)** the results of *DefluviitaleaceaeDefluviitaleaceae_UCG_011* genus; and **(C)** the results of this study suggest *Defluviitaleaceae* can increase anti-influenza H3N2 IgG levels to inhibit influenza which further prevents from subacute thyroiditis and hypothyroidism.

Firstly, variables related to hypothyroidism in FinnGen database are selected as outcome variables to understand the causal effect of *Defluviitaleaceae*. These factors include “thyroiditis,” “congenital iodine-deficiency syndrome,” “autoimmune hypothyroidism,” “postinfectious hypothyroidism,” “hypothyroidism due to medicaments and other exogenous,” and “other/unspecified hypothyroidism.” The results of thyroiditis are significant for both of *Defluviitaleaceae* family (OR 0.174, 95%CI 0.046–0.653, *P* = 0.029) and *Defluviitaleaceae_UCG_011* genus (OR 0.139, 95%CI 0.029–0.664, *P* = 0.043) (Figures 3A, B), while no significance is found in other outcome variables.

Then, MR analyses are performed between *Defluviitaleaceae* and three subtypes of thyroiditis in FinnGen database, which are “subacute thyroiditis” (family: OR 0.028, 95%CI 0.004–0.213, *P* = 0.007; genus: OR 0.018, 95%CI 0.002–0.194, *P* = 0.013) (Figures 3A, B), “autoimmune thyroiditis” (family: OR 0.982, 95%CI 0.459–2.104, *P* = 0.964; genus: OR 0.916, 95%CI 0.391–2.146, *P* = 0.840), and “unspecified thyroiditis” (family: OR 1.401, 95%CI 0.632–3.103, *P* = 0.406; genus: OR 2.254, 95%CI 0.926–5.487, *P* = 0.073) (Supplementary Table S8).

Therefore, the association of *Defluviitaleaceae* with mumps (family: OR 0.811, 95%CI 0.460–1.429, *P* = 0.469; genus: OR 0.791, 95%CI 0.419–1.492, *P* = 0.468) (Supplementary Table S8) and influenza (family: OR 0.818, 95%CI 0.676–0.989, *P* = 0.038; genus: OR 0.792, 95%CI 0.644–0.974, *P* = 0.027) (Figures 3A, B) are further explored, which are the common infections causing subacute thyroiditis. Because there are no summary-level GWAS data of coxsackievirus and adenovirus, the key proteins, coxsackievirus and adenovirus receptor, are analyzed as an alternative (family: OR 0.99,4 95%CI 0.818–1.209, *P* = 0.956; genus: OR 1.045, 95%CI 0.847–1.289, *P* = 0.679) (Supplementary Table S8). Although there is no causality found with coxsackievirus and adenovirus receptor in this study, it needs more direct evidence as proof.

Finally, MR analyses are performed between *Defluviitaleaceae* and antibody level of different subtypes of influenza. A significant increase of H3N2 IgG levels with *Defluviitaleaceae* family (OR 1.934, 95%CI 1.123–3.332, *P* = 0.017) was observed, while the result with *Defluviitaleaceae_UCG_011* genus is not significant (OR 1.675, 95%CI 0.953–2.943, *P* = 0.073) but still suggests an increase of the H3N2 antibody level (Figures 3A, B). Both the family and genus fail to find significance with H1N1 antibody level (family: OR 1.260, 95%CI 0.705–2.255, *P* = 0.435; genus: OR 1.210, 95%CI 0.651–2.249, *P* = 0.547) (Supplementary Table S8).

**Figure 4:**
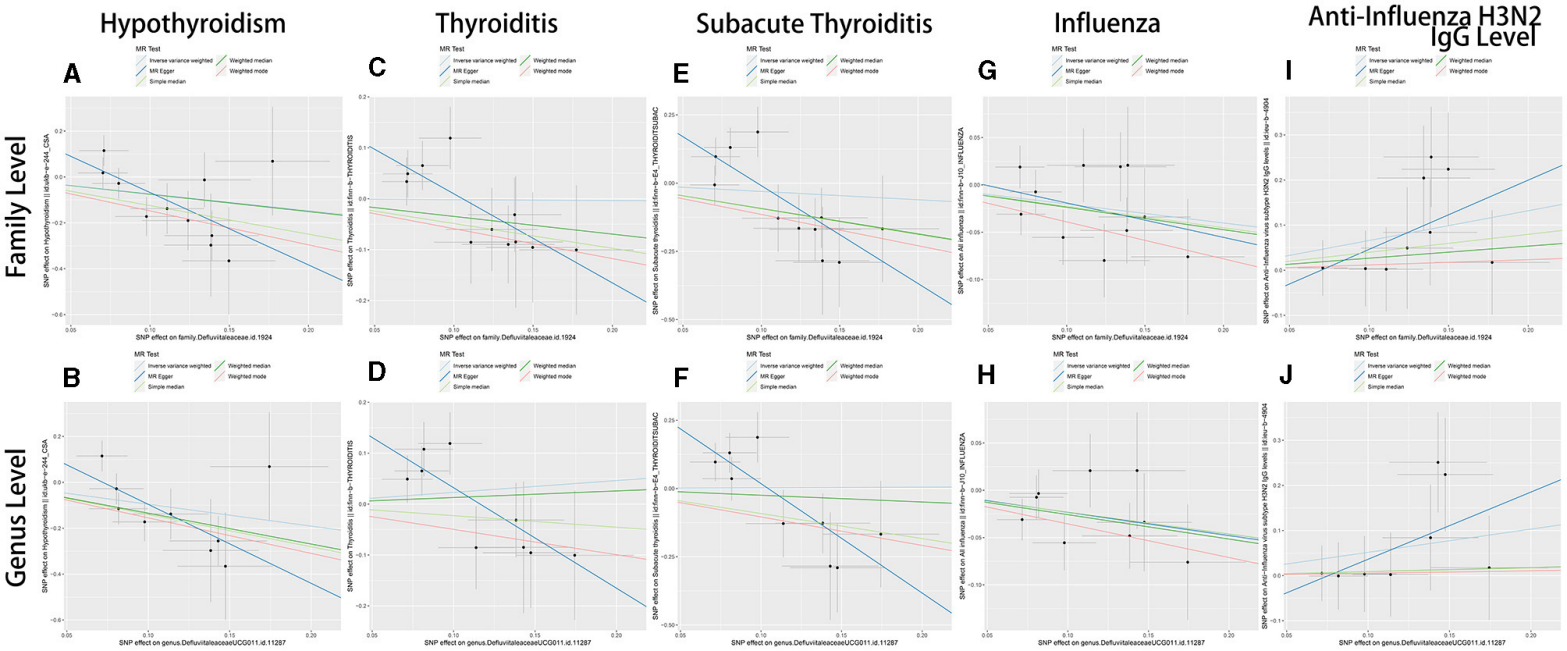
The scatter plots of MR results of *Defluviitaleaceae* family and *DefluviitaleaceaeDefluviitaleaceae_UCG_011* genus. **(A, B)** The results of hypothyroidism; **(C, D)** the results of thyroiditis; **(E, F)** the results of subacute thyroiditis; **(G, H)** the results of influenza; **(I, J)** the results of anti-influenza H3N2 IgG levels; **(A, C, E, G, I)** the results with *Defluviitaleaceae* family as exposure variable; and **(A, C, E, G, I)** the results with *DefluviitaleaceaeDefluviitaleaceae_UCG_011* genus as the exposure variable.

In sum, the results of the present study suggest that *Defluviitaleaceae* might be able to increase anti-influenza H3N2 IgG levels to decrease the occurrence of influenza. The decrease of influenza by *Defluviitaleaceae* prevents subacute thyroiditis and hypothyroidism (Figure 3C). Figure 4 shows the significant findings in the form of scatter plots.

As supplementary, forest plots (showing robustness of MR results) and leave-one-out plots (showing any outliers in IVs) of variables are displayed in Supplementary Figures S1, S2.

### Exploration of related mechanisms with enrichment analysis

There are 13 different SNPs as IVs of *Defluviitaleaceae*, of which 9 of them have corresponding genes, which are listed in Table 3A. Since only nine genes were used as input to perform enrichment analysis, the results with *P* < 0.1 were limited, and all of them are listed in Table 3B. The results show involvement in metabolic and immune fields, specifically with neutrophils, respiratory function tests, celiac disease, lipoproteins, and echocardiography results. In GO analysis, these genes are associated with cell adhesion. Although it is difficult to observe the association between cell adhesion and influenza, thyroiditis, and hypothyroidism, the enrichment results indicating immune system and neutrophils count are consistent with the finding of this study.

**Table 3:** The genes related to the instrument variables of *Defluviitaleaceae* and the significant enrichment analysis of these genes.

**A. Instrumental SNPs and corresponding genes**			
**SNPs**	**Gene**	**SNPs**	**Gene**
rs112893842	PTPRD	rs540220	LINC01501
rs1582238	SPAG17	rs55658617	DSCAM
rs17051335	None	rs72731813	SLC10A7
rs1908593	CDH20	rs9608282	SPECC1L
rs28696126	None	rs9725395	None
rs4344384	LOC124902439	rs2892880	None
rs4677103	LINC00870		
**B. Richment analysis of instrumental genes**			
**Category**	**Term**		***P*** **Value**
GAD_DISEASE_CLASS	METABOLIC		2.72E-02
GAD_DISEASE_CLASS	IMMUNE		4.05E-02
GAD_DISEASE	Celiac Disease		2.98E-03
GAD_DISEASE	Echocardiography		5.08E-03
GAD_DISEASE	Neutrophils		2.85E-02
GAD_DISEASE	Lipoproteins		3.75E-02
GAD_DISEASE	Lipoproteins, VLDL		4.83E-02
GAD_DISEASE	Respiratory Function Tests		7.43E-02
GOTERM_BP_DIRECT	GO:0007156~homophilic cell adhesion via plasma membrane adhesion molecules		4.39E-02
GOTERM_BP_DIRECT	GO:0007155~cell adhesion		7.97E-03

DefluviitaleaceaeDefluviitaleaceaeDefluviitaleaceae_UCG_011.

Since the nine SNP-related genes failed to find the involved pathway in KEGG pathway enrichment analysis, the transcription factors interacting with them were identified instead, which are listed in Supplementary Table S12A. The KEGG pathways of these transcription factors may give us some hints about the involved molecular pathways. The results can be observed in Supplementary Table S12B. It is worth noting that these indirect results are suggestive and have much weaker power. Since it is the indirect results of interacting transcription factors, the ranges get larger and are involved with the FoxO and JAK-STAT signaling pathway, immunity (e.g., Th17 cell differentiation), viral infection (e.g., measles and viral hepatitis), hormones (e.g., prolactin), metabolism, and cancer. The immunity, viral infection, and hormones are exactly consistent with the findings of the former part of this study.

## Discussion

Since the proposal of the “thyroid–gut–axis” concept (Lerner et al., 2017), quite a few studies have investigated the association between gut microbiota and thyroid-related diseases. Previous studies demonstrated that gut microbiota had an effect on thyroid disorders through the absorption of thyroid hormone-related nutrients, regulating the iodothyronine metabolism by enzymes (Virili and Centanni, 2017; Knezevic et al., 2020). Besides, gut microbiota might affect thyroid autoimmune diseases through interacting with host immune cells and secreting cytokines (Köhling et al., 2017; Shao et al., 2018). In this study, it was found that *Defluviitaleaceae*/*Defluviitaleaceae_UCG_011* were the protective factors for hypothyroidism, while *Bacteroidaceae*/*Bacteroides* were risk factors. Moreover, *Defluviitaleaceae* might prevent hypothyroidism induced by subacute thyroiditis by interacting with influenza.

In the human gut, Frimicutes and Bacteroidetes are the predominant phyla. Bacteroides are essential in the hydrolysis and fermentation of exogenous dietary fiber as well as endogenous mucins. Bacteroides have been realized with less colonization in patients with Grave's diseases (Ishaq et al., 2018). Moreover, the genus is also decreased in the intestines of mice with Grave's ophthalmopathy, which is the progression symptom of Grave's diseases (Masetti et al., 2018). Conversely, research also declared a reduction of Bacteroides in Hashimoto's thyroiditis patient fecal samples (Sawicka-Gutaj et al., 2022). Bacteroides were also discovered to be positively associated with anti-thyropreoxidase antibodies and negatively associated with thyroid-stimulating hormone levels (Sawicka-Gutaj et al., 2022; Fenneman et al., 2023). However, one species of Bacteroides, *Bacteroides fragilis*, is abundant in Hashimoto's thyroiditispatients. *Bacteroides fragilis* activates the NLRP3 expression, which is overexpressed in the thyroid tissue of Hashimoto's thyroiditis patients (Gong et al., 2021).

*Defluviitaleaceae*/*Defluviitaleaceae_UCG_011* was discovered by us to inhibit hypothyroidism. As a family in Clostridia*, Defluviitaleaceae*/*Defluviitaleaceae_UCG_011* seem to be a probiotic. Previous studies implied that the abundance of *Defluviitaleaceae*/*Defluviitaleaceae_UCG_011* was reduced in autoimmune-related diseases, such as rheumatoid arthritis, and systemic lupus erythematosus (Tong et al., 2019; Li et al., 2020). In addition, *Defluviitaleaceae_UCG_011* might play a protective role to reduce cardiac fibrosis (Du et al., 2022). However, the relationship between *Defluviitaleaceae*/*DefluviitaleaceaeDefluviitaleaceae_UCG_011* and thyroid-related diseases has not been investigated. The association between *Defluviitaleaceae*/*DefluviitaleaceaeDefluviitaleaceae_UCG_011* and different subtypes of hypothyroidism is further explored. *Defluviitaleaceae*/*DefluviitaleaceaeDefluviitaleaceae_UCG_011* was found to be a significantly protective factor for subacute thyroiditis. The potential mechanisms of these microbiota affecting thyroid function have not been investigated earlier. Viral infections including mumps and influenza were frequently regarded as a major cause of subacute thyroiditis (Desailloud and Hober, 2009). It was observed that influenza could cause subacute thyroiditis, such as H1N1 influenza infection (Dimos et al., 2010; Michas et al., 2014). Consistent with the findings of this study, the significant effect of *Defluviitaleaceae* to influenza antibodies was discovered. *Defluviitaleaceae* was likely to prevent subacute thyroiditis and hypothyroidism through influenza. *DefluviitaleaceaeDefluviitaleaceae_UCG_011*.

There have been many probiotics found using which homeostasis can be modeled to prevent from diseases (Mahmud et al., 2022). For *Defluviitaleaceae*, the results of enrichment analyses in this study indicate that it participates in individuals' metabolism and immunity, which are important fields for homeostasis. In the field of immune system, it is suggested to be associated with neutrophils (Table 3). In another study, *Defluviitaleaceae* was found to inhibit granulomatosis with polyangiitis through CD11c in granulocytes (Chen and Tang, 2023), most of which are neutrophils, which is consistent with the findings of this study. In addition, the indirect enrichment analyses with related transcription factors in this study (Supplementary Table S12) indicate the suggestive association with multiple viral infections, while influenza is also a viral disease. In the results related to metabolism in this study, *Defluviitaleaceae* seems to influence celiac disease and lipoproteins. There are also supporting studies despite which *Defluviitaleaceae* studies are limited. Cao et al. (2023) proved that *Defluviitaleaceae* were protective factors for visceral adipose tissue production, while Warbeck et al. (2021) reported that the abundance of *Defluviitaleaceae* was higher in the treatment group of celiac disease than controls. Although no significant results were found in the enrichment analyses of related genes of IVs, interacting transcription factors in Supplementary Table S12 were investigated as a suggestive reference, in the results of which the FoxO and JAK-STAT pathways are ranked at the top. The JAK-STAT signaling pathway is especially important to the immune system functions, including fighting infection, reinforcing barriers, and even cancer prevention (Hu et al., 2023a). Studies have proven the association between influenza and JAK-STAT signaling (Uetani et al., 2008). In relation to the relationship between JAK-STAT signaling pathway and thyroiditis, there is only one study that mentions the pathway participating in Grave's disease, which is another thyroiditis other than subacute thyroiditis (Li et al., 2024). However, the exploration of the mechanism of *Defluviitaleaceae* is indicative. More studies are warranted to further understand how *Defluviitaleaceae* can remodel the homeostasis of people.

As for the main method used in this study, MR is a method used in epidemiology and genetics to explore the causal relationships between modifiable exposures, intermediate factors, and outcomes. However, it can be challenging to explore molecular mechanisms and the potential drug target site if there are no adequate GWAS data. Some advanced prediction algorithms can be helpful for further investigation (Hu et al., 2023b). For example, researchers found a novel geometric deep learning and heterogeneous information network that can not only be useful in drug reposition (Zhao et al., 2022) but also in drug–target interactions (Zhao et al., 2023), which are both important in drug development and translational medicine. In the future, the application of advanced computational algorithms should facilitate an understanding of how *Defluviitaleaceae* remodels the individuals' homeostasis and contributes to treatment development related to the findings of this study.

There are limitations to this study. First, the summary-level GWAS data used in this study cannot be used to perform stratified analyses by covariates, such as age and sex. Second, the findings of this study are based on two-sample MR methods, which are used to investigate linear causal associations. Any non-linear effects of the gut microbiota on the outcome variables involved in this study are unable to be assessed. Third, the exploration of molecular mechanism in this study is still superficial and might be difficult to extrapolate to other diseases. Further research, especially bacterial studies *in vivo*, are warranted to validate the findings and further understand how *Defluviitaleaceae* regulates homeostasis and prevents diseases. Finally, the results only indicate the key role of *Defluviitaleaceae* family and *DefluviitaleaceaeDefluviitaleaceae_UCG_011* genus. Since the data source of the gut microbiota does not include any data of specific bacterial species, in this study, we were unable to explore the effect of different species in *DefluviitaleaceaeDefluviitaleaceae_UCG_011* genus. Therefore, further *Defluviitaleaceae* supplementation trials are needed in the future to verify the relationship between *Defluviitaleaceae* and the clinical pathway of “Influenza–Subacute Thyroiditis–Hypothyroidism.”

## Data availability statement

The original contributions presented in the study are included in the article/Supplementary material, further inquiries can be directed to the corresponding authors.

## Author contributions

XZ: Conceptualization, Formal analysis, Methodology, Writing – original draft. P-HL: Conceptualization, Formal analysis, Methodology, Writing – original draft. DW: Conceptualization, Formal analysis, Methodology, Writing – original draft. HLi: Validation, Writing – review & editing. XK: Validation, Writing – review & editing. GZ: Validation, Writing – review & editing. YZhao: Validation, Writing – review & editing. JL: Validation, Writing – review & editing. WW: Validation, Writing – review & editing. YZhan: Funding acquisition, Supervision, Writing – review & editing. Z-HL: Funding acquisition, Supervision, Writing – review & editing. HLu: Funding acquisition, Supervision, Writing – review & editing.
