# Supplementary material for: Causal effect of gut microbiota of Defluviitaleaceae on the clinical pathway of “Influenza–Subacute Thyroiditis–Hypothyroidism”
Source: Front Microbiol. 2024 Feb 26;15:1354989. doi: 10.3389/fmicb.2024.1354989 (PMC10929266; doi:10.3389/fmicb.2024.1354989)
Supplement: Supplementary file 1 [file Data_Sheet_1.ZIP › SupplementaryMaterials/Legends for supplementary materials.docx]

**Figure S1.** The forest plots showing robustness of the significant MR results. **(a, b)** Results of hypothyroidism; **(c, d)** Results of thyroiditis; **(e, f)** Results of subacute thyroiditis; **(g, h)** Results of influenza; **(i, j)** Results of anti-influenza H3N2 IgG levels; **(a, c, e, g, i)** Results with Defluviitaleaceae family as exposure variable; **(a, c, e, g, i)** Results with Defluviitaleaceae_UCG_011 genus as exposure variable.

**Figure S2.** The leave-one-out plots showing any outliers of instrument variables. **(a, b)** Results of hypothyroidism; **(c, d)** Results of thyroiditis; **(e, f)** Results of subacute thyroiditis; **(g, h)** Results of influenza; **(i, j)** Results of anti-influenza H3N2 IgG levels; **(a, c, e, g, i)** Results with Defluviitaleaceae family as exposure variable; **(a, c, e, g, i)** Results with Defluviitaleaceae_UCG_011 genus as exposure variable.

**Table S1.** Summary of data source

**Table S2.** Details of SNPs selected as IVs of gut microbiota.

**Table S3.** The results of causality evaluated in MR analyses, including results of all taxa and all MR methods.

**Table S4.** Results of heterogeneity and directional horizontal pleiotropy of single nucleotide polymorphism effect of all taxa on hypothyroidism.

**Table S5.** Heterogeneity and directional horizontal pleiotropy of single nucleotide polymorphism of taxa of gut microbiota which have significant causal estimates of primary methods.

**Table S6.** MR Steiger directionality test indicated that all taxa are upstream of hypothyroidism.

**Table S7.** Details of SNPs selected as IVs in investigating causal effects of Defluviitaleaceae family and Defluviitaleaceae_UCG_011 genus on hypothyroidism.

**Table S8.** The results of causality evaluated in MR analyses, including results between Defluviitaleaceae family and Defluviitaleaceae_UCG_011 genus, and all outcome variables used in this study.

**Table S9.** Results of heterogeneity and directional horizontal pleiotropy of single nucleotide polymorphism effect of Defluviitaleaceae family and Defluviitaleaceae_UCG_011 genus on hypothyroidism, thyroiditis, sub thyroiditis, influenza, and anti-influenza H3N2 IgG level.

**Table 10.** Heterogeneity and directional horizontal pleiotropy of single nucleotide polymorphism used in investigating causal effects of Defluviitaleaceae family and Defluviitaleaceae_UCG_011 genus on hypothyroidism, thyroiditis, sub thyroiditis, influenza, and anti-influenza H3N2 IgG level.

**Table S11.** MR Steiger directionality test indicated that all results of Defluviitaleaceae family and Defluviitaleaceae_UCG_011 genus have no reverse causation.

**Table S12.** The genes of transcription factors interacting with SNP-related gene sof Defluviitaleaceae and the corresponding pathway enrichment analysis results.
