# Supplementary material for: Causal effect of gut microbiota of Defluviitaleaceae on the clinical pathway of “Influenza–Subacute Thyroiditis–Hypothyroidism”
Source: Front Microbiol. 2024 Feb 26;15:1354989. doi: 10.3389/fmicb.2024.1354989 (PMC10929266; doi:10.3389/fmicb.2024.1354989)

**Introduction of Analytic Methods of Mendelian Randomization (MR) in this study**

All analytic methods used in this study can be directly performed with “two-sampleMR” package on R, which is widely used in MR studies. This note is to provide a brief introduction of each method.

**Inverse variance weighted (IVW)**

The IVW method synthesizes the effects of multiple genetic variants associated with the exposure on the outcome, typically through a meta-analysis framework. It estimates the causal effect by combining the individual genetic variant-exposure associations with their corresponding associations with the outcome. The weights assigned to each genetic variant in the IVW method are usually based on the inverse of their variance, hence the name "inverse variance weighted."

The IVW method assumes that all genetic variants used as instruments satisfy three key assumptions of Mendelian Randomization. The IVW method assumes no heterogeneity or horizontal pleiotropy in the causal effect estimates across the genetic variants.

Researchers often complement the IVW method with sensitivity analyses to detect and account for potential violations of these assumptions and to provide more robust causal inference in Mendelian Randomization studies.

If the association of the *j* th variant is as:

$\beta_{Yj}=\alpha_{j}+\theta\beta_{Xj}$

($\alpha_{j}$ is the pleiotropic effect of the genetic variant on the outcome, and $\theta\beta_{Xj}$ is the causal effect via the exposure. $\theta$ is the causal effect.)

The weighted average of the variant-specific causal estimates of the IVW estimate is like following:

$\hat{\theta}_{IVW}=\frac{\sum_{j} \hat{\theta}_{j}{se\left( \hat{\theta}_{j} \right)}^{-2}}{\sum_{j} {se\left( \hat{\theta}_{j} \right)}^{-2}}$=$\frac{\sum_{j} \hat{\beta}_{Yj}\hat{\beta}_{Xj}{se\left( \hat{\beta}_{Yj} \right)}^{-2}}{\sum_{j} {\hat{\beta}_{Xj}}^{2}{se\left( \hat{\beta}_{Yj} \right)}^{-2}}$

The (fixed-effect) standard error of the IVW estimate is:

$se\left( \hat{\theta}_{IVW} \right)=\sqrt{\frac{1}{\sum_{j} {\hat{\beta}_{Xj}}^{2}{se\left( \hat{\beta}_{Yj} \right)}^{-2}}}$

**MR Egger**

MR Egger specifically addresses potential violations of key assumptions in MR, such as horizontal pleiotropy, which occurs when genetic variants associated with the exposure also affect the outcome through pathways unrelated to the exposure. MR Egger also provides a way to assess and correct for bias due to pleiotropy.

The MR Egger method is performed similarly to the IVW method, except that the regression model contains an intercept term of *θ_0_*. The estimate of the slope parameter *θ* is the MR Egger estimate.

$$\hat{\beta}_{Yj}=\theta_{0}+\theta\hat{\beta}_{Xj}+\varepsilon_{j}, \varepsilon_{j}\sim N(0,{se\left( \hat{\beta}_{Yj} \right)}^{2})$$

The MR Egger method involves regression analysis of the genetic variants' associations with the exposure and outcome, allowing for an intercept term that captures potential pleiotropy. By examining the intercept, researchers can gain insights into the presence and direction of pleiotropy. If the intercept is non-zero, it suggests the presence of pleiotropy.

The MR Egger does not have such strong assumptions like IVW method. But, instead, the results of MR Egger are also less efficient than IVW.

**Weighted Median and Simple Median**

The median method calculates the causal estimate by directly taking the median of the individual genetic variant-exposure effect estimates and their associations with the outcome. Provided that the majority of the genetic variants are valid instrumental variables (IVs), this means that the median of the ratio estimates will tend towards the true causal effect. In a finite sample size, estimates from invalid IVs will still influence the median estimate, but they will have far less influence than for the IVW estimate.

In the simple median method, all genetic variants receive equal weight in the analysis. A weighted version of the median method can also be calculated. In the weighted median method, we consider an empirical distribution in which each variant receives a weight (the same weight as in the IVW method). Estimates are ranked in order, and the weighted median estimate is taken as the estimate at the median of the distribution.

Standard errors and confidence intervals for the median method are constructed by parametric bootstrapping of the genetic association estimates, making a normal assumption for the estimate. We refer to the assumption that over 50% of the variants are valid IVs as the “majority valid” assumption.

**Weighted Mode**

Suppose that 40% of the variants have estimates tending towards one value, 10% towards a second value, 10% towards a third value, 10% towards a fourth value, and so on. These values cannot all be the true causal effect of the exposure on the outcome. It seems plausible to assert that the value supported by 40% of the variants carries more substantial evidence as the true causal effect.

The assumption of "plurality validity" suggests that among the various values derived from ratio estimates in large samples, the true causal effect is the value taken for the largest number of genetic variants. This assumption allows the true causal effect to be identified in cases where less than 50% of variants are valid IVs.

This assumption is exploited by the mode-based estimation method. As no two ratio estimates will be identical in finite samples, it is not possible to take the mode of the ratio estimates directly. In the mode-based estimation method, a normal distribution is created for each genetic variant, centered around its ratio estimate. The width of this distribution relies on a bandwidth parameter and, in the weighted mode-based estimation approach, the precision of the ratio estimate. A smoothed density function is then obtained by summing these normal densities. The maximum of this distribution is the causal estimate. Confidence intervals are established through parametric bootstrapping, assuming a normal distribution for the causal estimate.

**Summary of Methods above**

| **Method** | **Strength** | **Reference** |
| --- | --- | --- |
| Inverse variance weighted (IVW) | (Need IVs to be all valid and no horizontal pleiotropy)  Most efficient, with greatest statistical power) | Burgess S et al. Mendelian randomization analysis with multiple genetic variants using summarized data. Genetic Epidemiol. 2013; 37(7):658-65. |
| MR Egger | Robust to pleiotropy  (But less efficient) | Bowden J et al. Mendelian randomization with invalid instruments: effect estimation and bias detection through Egger regression. International Journal of Epidemiology 2015; 44:512-525. |
| Weighted median/Simple median | (Need majority of IVs valid)  Robust to outliers | Bowden J et al. Consistent Estimation in Mendelian Randomization with Some Invalid Instruments Using a Weighted Median Estimator. Genet Epidemiol 2016; 40:304-14. |
| Weighted Mode/Mode-based estimation | (Need plurality of IVs valid)  Robust to outliers  (But less efficient) | Hartwig FP et al. Robust inference in summary data Mendelian randomization via the zero modal pleiotropy assumption. International Journal of Epidemiology 2017; 46:1985-1998. |

**Selection of Primary Methods in this study**

According to the traits of methods summarized above, the selection of primary methods is performed following the flow chart below.


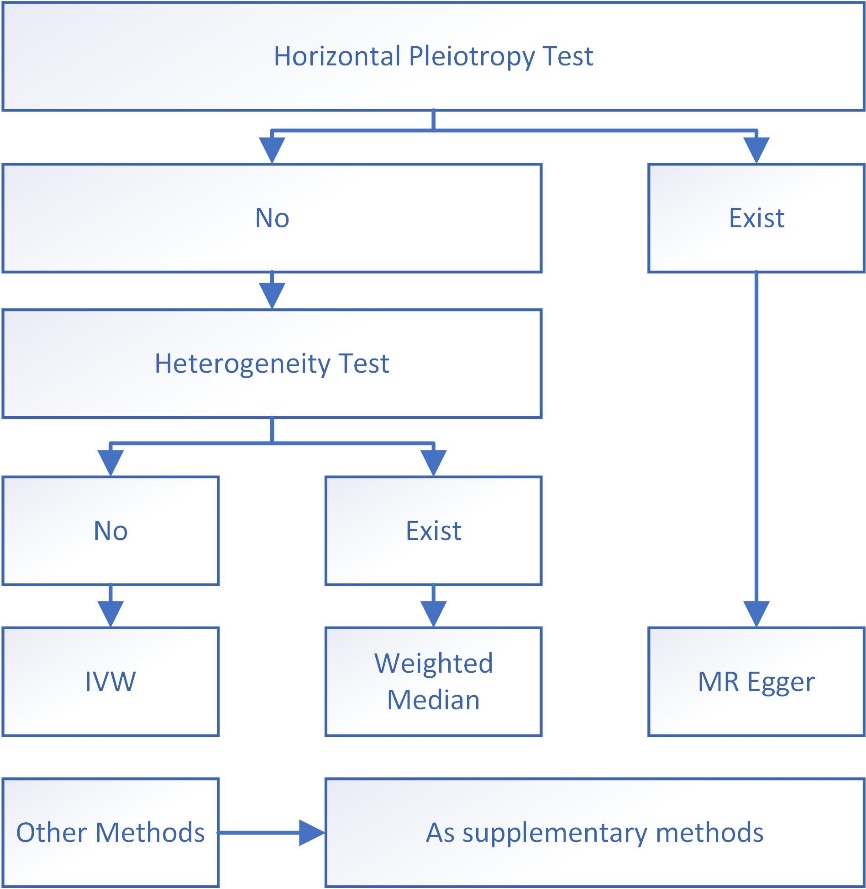

Supplement: Supplementary file 1 [file Data_Sheet_1.ZIP › SupplementaryMaterials/SupplementaryNote.docx]
