# Supplementary figures and images for: Causal effect of gut microbiota of Defluviitaleaceae on the clinical pathway of “Influenza–Subacute Thyroiditis–Hypothyroidism”
Source: Front Microbiol. 2024 Feb 26;15:1354989. doi: 10.3389/fmicb.2024.1354989 (PMC10929266; doi:10.3389/fmicb.2024.1354989)

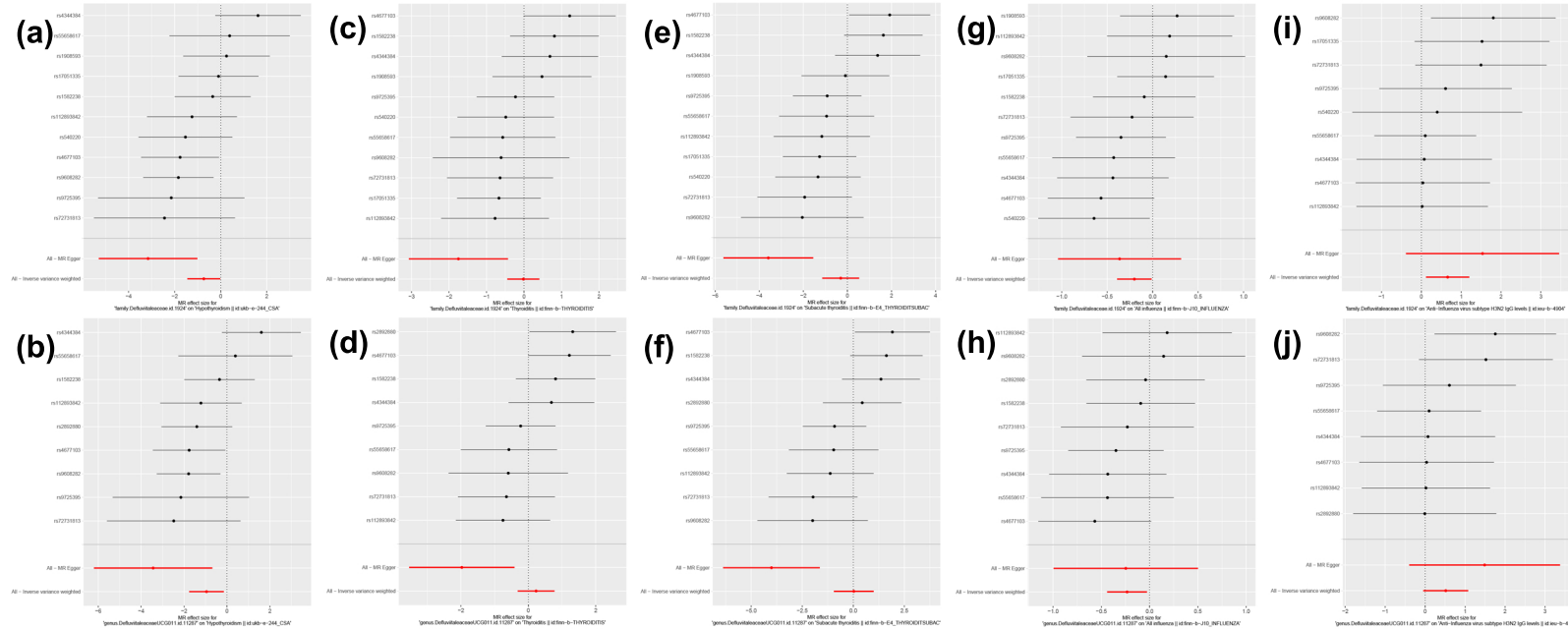

Supplement: Supplementary file 1 [file Data_Sheet_1.ZIP › SupplementaryMaterials/FigureS1.pdf]

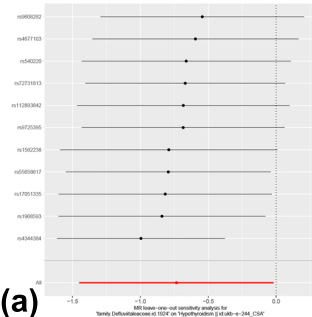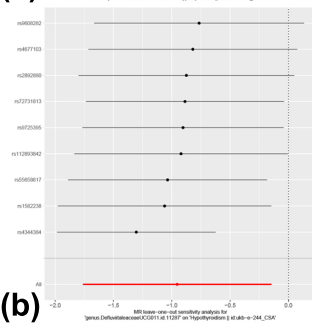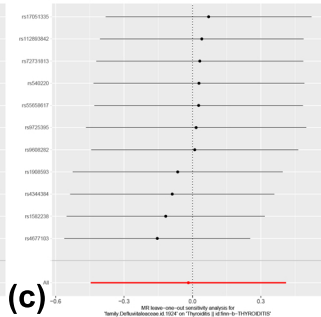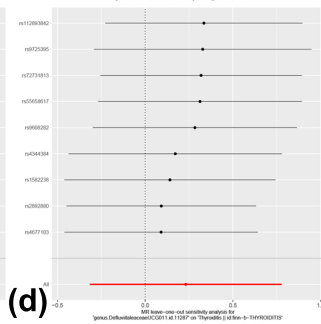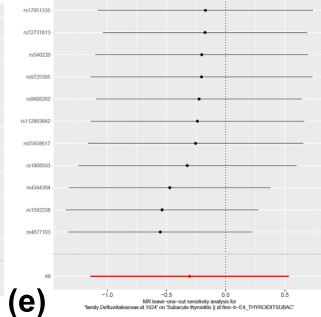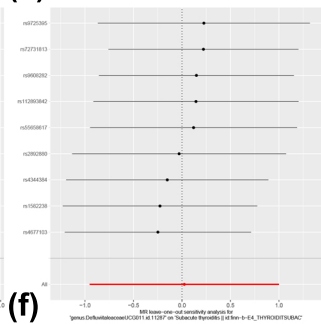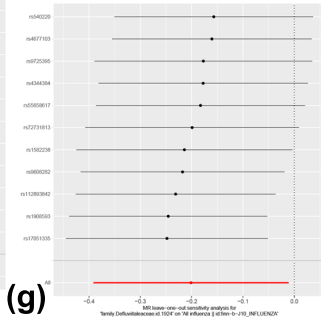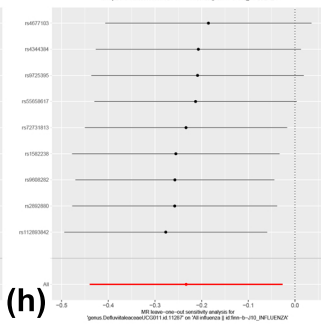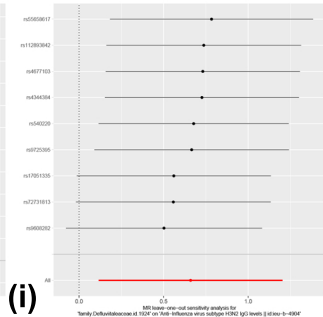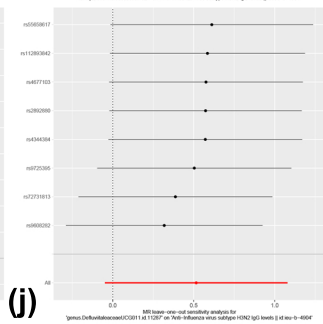

Supplement: Supplementary file 1 [file Data_Sheet_1.ZIP › SupplementaryMaterials/FigureS2.pdf]
